# First Detection of *Cryptosporidium* spp. in Migratory Whooper Swans (*Cygnus cygnus*) in China

**DOI:** 10.3390/microorganisms8010006

**Published:** 2019-12-18

**Authors:** Ke Wang, Azhar Gazizova, Yuexin Wang, Kaihui Zhang, Yifan Zhang, Yankai Chang, Yuan Cui, Yuxi Zhang, Sumei Zhang, Longxian Zhang

**Affiliations:** 1 College of Animal Science and Veterinary Medicine, Henan Agricultural University, Zhengzhou 450046, China; 18838988962@163.com (K.W.); azhar.gazizova13@gmail.com (A.G.); 18737615649@163.com (Y.W.); zkh15886723100@163.com (K.Z.); changyankai369@163.com (Y.C.); 2 Sanmenxia Management Office of Henan Yellow River Wetland National Nature Reserve, Sanmenxia 472000, China; zyf2629@126.com (Y.Z.); onlycuiyuan@126.com (Y.C.); 3 Sanmenxia Swan Lake National Urban Wetland Park Management Office, Sanmenxia 472001, China; dakaihe2012@163.com

**Keywords:** *Cryptosporidium*, whooper swans, zoonotic

## Abstract

*Cryptosporidium* is an important protozoan parasite that can cause gastrointestinal diseases in humans and that also causes respiratory and gastrointestinal diseases in birds. In this study, we investigated the occurrence of *Cryptosporidium* species in migratory whooper swans in China. Fecal samples (*n* = 467) from whooper swans were collected from Sanmenxia Swan Lake National Urban Wetland Park, China. The samples were analyzed for *Cryptosporidium* species and genotypes with PCR along a sequence analysis of the small subunit rRNA. *Cryptosporidium* was detected in eight of the 467 (1.7%) samples. The analysis of the small subunit rRNA sequence data revealed two zoonotic species (*Cryptosporidium parvum* and *Cryptosporidium andersoni*) and one genotype (*Cryptosporidium* goose genotype II). These are the first data on the positive rate of *Cryptosporidium* spp. in whooper swans in China, and they suggest that whooper swans can harbor the zoonotic species *C. parvum* and *C. andersoni* in China.

## 1. Introduction

*Cryptosporidium* species are apicomplexan parasites and have a broad range of vertebrate hosts (including mammals and birds) [1,2]. The typical clinical symptom of *Cryptosporidium* infection in humans is watery diarrhea, which can lead to death in immunocompromised patients [3]. Symptoms such as diarrhea, cough, and dyspnea, as well as high morbidity and mortality may be observed in *Cryptosporidium*-infected birds [4], which can excrete feces containing *Cryptosporidium* oocysts into recreational and drinking water. Humans can acquire *Cryptosporidium* oocysts by ingesting contaminated food and water [5] or by direct contact with infected birds [6].

Based on molecular methods and morphological data, 38 *Cryptosporidium* species and more than 70 *Cryptosporidium* genotypes have been identified so far in various hosts [7]. Birds are mainly infected by *Cryptosporidium baileyi*, *Cryptosporidium meleagridis*, *Cryptosporidium galli*, and *Cryptosporidium avium* [8], but several other *Cryptosporidium* species and genotypes have been reported in birds in other studies, including *Cryptosporidium parvum*, *Cryptosporidium andersoni*, *Cryptosporidium hominis*, *Cryptosporidium muris*, *Cryptosporidium* goose genotypes (I–IV), a *Cryptosporidium* duck genotype, and *Cryptosporidium* avian genotypes (I–IV) [9,10].

The whooper swan (*Cygnus cygnus*) is a large, predominantly herbivorous, migratory waterfowl in the family Anatidae. It winters on freshwater lakes, brackish lagoons, coastal bays, low-lying coastal agricultural land and wet pastures. The whooper swan migrates along the middle and upper reaches of the Yellow River and its tributaries in spring after moving out of the Sanmenxia wetland, and it arrives at its breeding grounds in central and western Mongolia after flying over the Yinshan Mountains of China [11]. More than 10,000 whooper swans spend the winter in Sanmenxia Swan Lake National Urban Wetland Park every year, attracting many tourists. Tourists feed whooper swans in the Sanmenxia wetland, which is close to residential areas. Their excrement is scattered in public areas, including on grass and in water. Whooper swans reportedly migrate through at least 40 water bodies and lakes, posing a potential threat to public health [12].

Over 20 pathogenic microorganisms have been identified in swans to date, including avian influenza virus, *Toxoplasma gondii*, Microsporidia and *Giardia* [13,14,15,16,17]. However, the positive rate of *Cryptosporidium* and the number of *Cryptosporidium* species in whooper swans are unknown. Therefore, the aims of this study were to investigate the positive rate and species/genotypes of *Cryptosporidium* in whooper swans in China and to evaluate the potential threat of *Cryptosporidium* infection they pose to public health.

## 2. Materials and Methods

### 2.1. Ethics Approval

This study was conducted in accordance with the Law on the Protection of Wildlife adopted in 1988 in the People’s Republic of China. The research protocol was examined and approved by the Research Ethics Committee of Henan Agricultural University (Approval No. IRC-HENAU-20180919-05). Permission was obtained from all the managers of the study site before the fecal samples were collected.

### 2.2. Sample Collection

**Figure 1:**
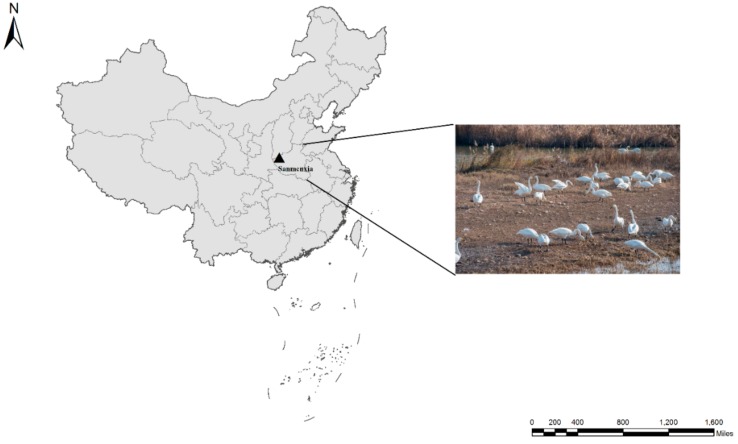
Location of the city (▲) in which the samples were collected.

In total, 467 fecal specimens were collected from whooper swans in Sanmenxia Swan Lake National Urban Wetland Park in Sanmenxia city in the middle reaches of the Yellow River, China (Figure 1). In order to investigate the variation of the positive rate of *Cryptosporidium* in whooper swans over time and to further clarify the source of *Cryptosporidium*, we divided the sampling into three periods. Whooper swans arrive in the Sanmenxia wetland in November. Of the 467 samples, 237 were collected in November 2018, 161 were collected in December 2018, and 69 were collected in March 2019. The fresh fecal samples were collected immediately after defecation with a sterile disposal latex glove, and they were stored in 2.5% potassium dichromate at 4 °C before DNA extraction. To avoid the duplicated samples, each sample was collected in a short time with the help of the park staff.

### 2.3. DNA Extraction

The genomic DNA was extracted from the fecal samples with the commercial E.Z.N.A.^®^ Stool DNA Kit (Omega Bio-Tek Inc., Norcross, GA, USA) according to the manufacturer’s instructions. The extracted DNA samples were stored at −20 °C before their PCR analysis for *Cryptosporidium*.

### 2.4. Cryptosporidium PCR Assays

*Cryptosporidium* species were detected with nested PCR targeting a fragment (approximately 830 bp) of the small subunit (SSU) rRNA gene. Xiao’s method, which was based on the small subunit rRNA sequence of *Cryptosporidium*, was used to design the nested PCR primers for the detection of *Cryptosporidium* [18,19]. The amplification was performed in 25 µL reaction mixtures. The first reaction mixture contained 1 μL of extracted DNA. The second reaction mixture contained 1 µL of the first PCR amplification product, and this was diluted 10-fold with sterile, double-distilled water. The KOD Plus DNA polymerase (Toyobo Co., Ltd., Osaka, Japan) was used for all PCR amplification. The second amplification products were examined with 1% (*w*/*v*) agarose gel electrophoresis after staining with DNA Green (Solarbio, Beijing, China). All PCRs were performed in triplicate, and both positive and negative control PCRs were included with each run.

### 2.5. Sequencing and Sequence Analysis

All positive amplification products were bidirectionally sequenced on an ABI PRISM™ 3730xl DNA Analyzer (Applied Biosystems, Foster City, CA, USA) along with the BigDye Terminator v3.1 Cycle Sequencing Kit (Applied Biosystems). To determine the *Cryptosporidium* genotypes, ClustalX 2.1 (http://www.clustal.org/) was used to align each sequence with a reference sequence downloaded from GenBank. The phylogenetic relationships were determined with the neighbor-joining (NJ) algorithm in MEGA X (http://www.megasoftware.net/). On the phylogenetic tree, the lengths of the branches represent the evolutionary distances between genotypes, which were calculated according to the Kimura 2-parameter model. The accuracy of the tree was assessed with the bootstrap method with 1000 replicates.

## 3. Results

### 3.1. Cryptosporidium Positive Rate

According to the SSU-rRNA-based PCR, eight (1.7%) of the 467 fecal samples in this study were positive for *Cryptosporidium*. The positive rate of *Cryptosporidium* in the first sample group (2.9%, 7/237) was significantly higher than that in the second (0.62%, 1/161) and third sample groups (0%, 0/69; Table 1).

All PCR-positive samples were successfully sequenced. The nucleotide sequence analysis of the 18S rRNA revealed three distinct *Cryptosporidium* species or genotypes: *C. parvum*, *C. andersoni*, and *Cryptosporidium* goose genotype II (Table 1).

### 3.2. Phylogenetic Analysis of Cryptosporidium Isolates from Whooper Swans

**Figure 2:**
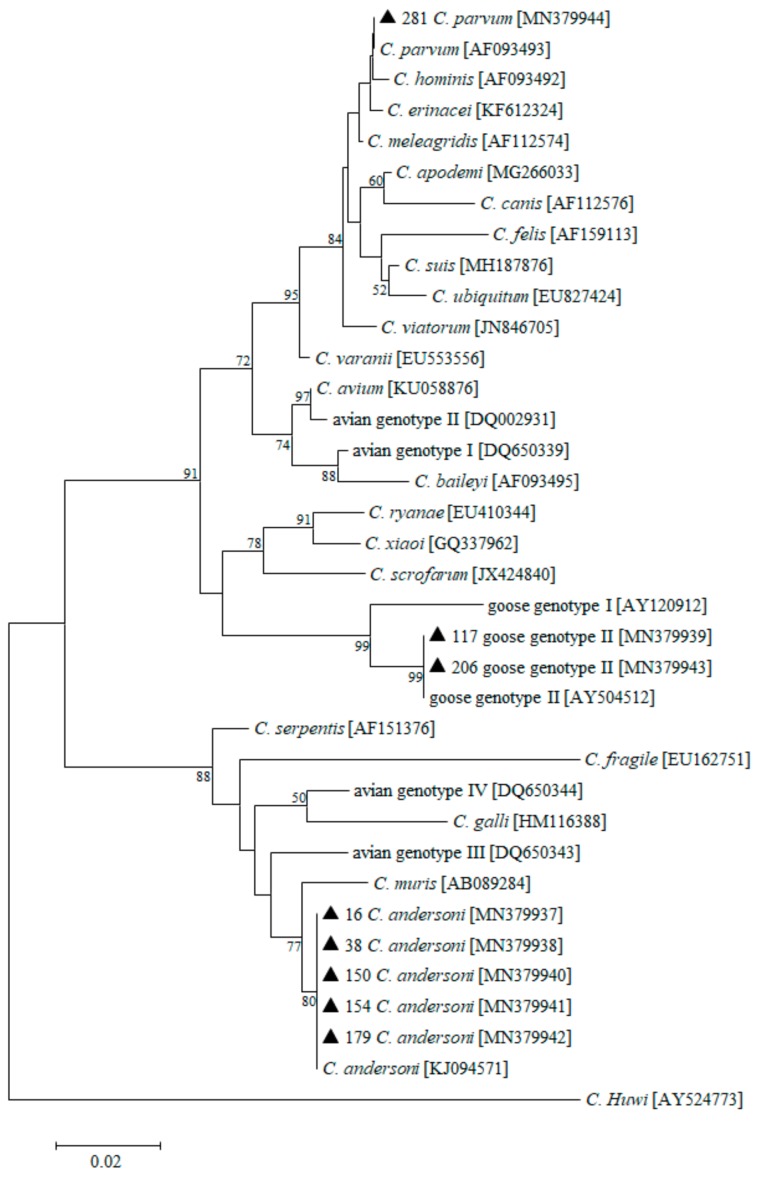
Neighbor-joining tree based on small subunit (SSU) rRNA gene sequences of *Cryptosporidium*. GenBank accession numbers are shown in parentheses after the isolate identifiers. Numbers on the branches are percentage bootstrap values >50% that were calculated from 1000 replicates. Genotypes marked with filled triangles are known genotypes identified in the present study.

An NJ tree was constructed from the 18S rRNA gene sequences that were isolated in this study and representative sequences from GenBank. The sequence from one fecal sample was identical to that of *C. parvum* (accession no. MN379944) and shared 100% identity with an isolate (AF093493) from a calf in the USA. *C. andersoni* (accession nos MN379937, MN379938, MN379940, MN379941, and MN379942) was detected in five specimens from whooper swans, with nucleotide sequences identical to the reference sequence (KJ094571) that was isolated from white yaks in China. The two remaining isolates were identical to that of *Cryptosporidium* goose genotype II (accession nos MN379939 and MN379943), and it shared 100% identity with an isolate from Canada geese (*Branta canadensis*) (AY504512) (Figure 2).

## 4. Discussion

This study is the first to investigate the positive rate and genotypes of *Cryptosporidium* spp. in whooper swans in China. The overall positive rate of *Cryptosporidium* was 1.7% (8/467) in these whooper swans, a rate which is similar to that previously reported in wild birds in Álava (2.3%, 6/265) and Algeria (2.8%, 2/71) [20,21]. However, in this study, the infection rate of *Cryptosporidium* was lower than those in free-living wild birds in Hungary (5.8% 6/103), Brazil (6.6% 16/242), and Spain (8.3% 36/433) [22,23,24]. Other studies have also reported much higher infection rates of *Cryptosporidium* in wild birds than this study, e.g., in wild captive psittacines in Brazil (10.64% 5/47), Java sparrows (*Lonchura oryzivora*) in northern China (13.42% 47/350), and North American red-winged blackbirds (*Agelaius phoeniceus*) (17.1% 12/70), and Canada geese (23.4% 49/209) in Ohio and Illinois [25,26,27,28]. Previous studies recorded *Cryptosporidium* infection rates in domestic birds of 2.3%–4.86% in Brazil [29,30,31] and 0.82%–8.1% in China [32,33,34,35,36]. These variations in the positive rate of *Cryptosporidium* in different studies may be attributable to population densities, feeding habits, and climate. Interestingly, the inconsistence in the positive rate of the three sample groups suggests that *Cryptosporidium* detected here might be from the origin or route of the migration instead of the sampling site.

Our study shows that two *Cryptosporidium* species and one genotype were identified in the whooper swans, namely *C. parvum*, *C. andersoni,* and *Cryptosporidium* goose genotype II. The zoonotic species *C. parvum* was detected in the whooper swans in this study, and this species has also been found in the feces of migratory Canada geese (*Branta canadensis*) in other studies [25,37]. *C. parvum* is responsible for most cases of cryptosporidiosis in humans and preweaned calves in China [38,39]. Previous reports have demonstrated that migratory herbivorous birds can acquire *Cryptosporidium* oocysts by ingesting undigested plant material in cattle feces. Other studies have reported that *Cryptosporidium* is found in wild birds, including the mute swan, white stork, carrion crow, mandarin duck, common merganser, rook, sparrowhawk, common buzzard, black kite, and honey buzzard [16,22]. *C. parvum* has been reported in domestic birds throughout the world, including Bengalese finches, psittacines, and cockatiels [31,40,41].

Recent studies have reported that *C. andersoni* can infect the quail-crested wood partridge [42], ostrich [43], and Canada goose [44]. However, there have been no reported cases of *C. andersoni* infection in domestic birds. It is well known that *C. andersoni* is the most common *Cryptosporidium* species in post-weaned calves and adult cattle, a fact which has been confirmed in China and Mongolia [45,46], and this species is known to infect humans [47].

In the present study, we detected *Cryptosporidium* goose genotype II in the feces of whooper swans. Natural infections of *Cryptosporidium* goose genotype II were first reported in Canada geese in America [25]. However, there have been no reports of infections of this genotype in other animals (including humans). Therefore, the public-health significance of this genotype remains unclear, and whether the whooper swan is a new host of this genotype still needs more investigation. Interestingly, four common species of *Cryptosporidium* in birds—*C. baileyi*, *C. meleagridis*, *C. galli*, and *C. avium*—were not identified in this survey. In addition, the dominant species/genotypes were different from each other in the three sample groups. Together with the above-discussed information, these data further indicate that the *Cryptosporidium* detected in the first sample groups might not originate from the sampling site but from the origin or route of the migration.

## 5. Conclusions

The presence of *C. parvum*, *C. andersoni*, and *Cryptosporidium* goose genotype II suggests that whooper swans may serve as transport hosts for *Cryptosporidium spp.* The inconsistence of species/genotypes detected in the three sample groups also suggests that whooper swans might play a role in the cross-regional transmission of *Cryptosporidium*, and this is the first report of *Cryptosporidium* in whooper swans.

## Figures and Tables

**Table 1:** *Cryptosporidium* genotypes in whooper swans in China.

Sampling	Sample Size	No. Positive Samples	Species and/or Genotype
1st (November 2018)	237	7	*C. andersoni* (5), *Cryptosporidium* goose genotype II (2)
2nd (December 2018)	161	1	*C. parvum* (1)
3rd (March 2019)	69	0	None
